# Emergence, Evolution and Scaling of Online Social Networks

**DOI:** 10.1371/journal.pone.0111013

**Published:** 2014-11-07

**Authors:** Le-Zhi Wang, Zi-Gang Huang, Zhi-Hai Rong, Xiao-Fan Wang, Ying-Cheng Lai

**Affiliations:** 1 School of Electrical, Computer and Energy Engineering, Arizona State University, Tempe, Arizona, United States of America; 2 School of Physical Science and Technology, Lanzhou University, Lanzhou, China; 3 Web Sciences Center, School of Computer Science and Engineering, University of Electronic Science and Technology of China, Sichuan, China; 4 Department of Automation, Shanghai Jiao Tong University, Shanghai, China; 5 Department of Physics, Arizona State University, Tempe, Arizona, United States of America; Tianjin University, China

## Abstract

Online social networks have become increasingly ubiquitous and understanding their structural, dynamical, and scaling properties not only is of fundamental interest but also has a broad range of applications. Such networks can be extremely dynamic, generated almost instantaneously by, for example, breaking-news items. We investigate a common class of online social networks, the user-user retweeting networks, by analyzing the empirical data collected from Sina Weibo (a massive twitter-like microblogging social network in China) with respect to the topic of the 2011 Japan earthquake. We uncover a number of algebraic scaling relations governing the growth and structure of the network and develop a probabilistic model that captures the basic dynamical features of the system. The model is capable of reproducing all the empirical results. Our analysis not only reveals the basic mechanisms underlying the dynamics of the retweeting networks, but also provides general insights into the control of information spreading on such networks.

## Introduction

Online social networks have become an indispensable part of our modern society for obtaining and spreading information. A piece of breaking news can activate a corresponding online social network, through which the news topic can spread rapidly to many individuals. By its very nature an online network is necessarily time dependent, growing rapidly in size initially as the news spreads out and saturating after certain amount of time. Since online social networks concerning certain topics can be active for only a transient period of time, they are *extremely dynamic*, which is quite distinct from, *e.g.*, the typical networks studied in the literature where they can be regarded as stationary with respect to the time scale of typical dynamical processes supported. A question of interest is whether there are general rules underlying the evolution of online social networks. A viable approach to addressing this question is to analyze large empirical data sets that are becoming increasingly accessible [1]. In fact, recent years have witnessed a growing research interest in online social network systems. There have been efforts in issues such as network and opinion co-evolution [2], users participation comparison for topics of current interest [3], information diffusion patterns in different domains [4], [5], the dynamics of users' activity across topics and time [6], [7], users behavior modeling on networks [8], [9], popular topic-style analysis in the Twitter-like social media [10]–[12], users influence in social networks [13], and language geography studies of Twitter data set [14].

In this paper, we aim to uncover the fundamental mechanisms underpinning the dynamical evolution of online social networks through empirical-data analysis. Our data come from Sina Weibo, a twitter-like microblogging social network medium in China. The appealing features of the data include wide publicity, real-time availability of information, and message compactness. Similar to Twitter, Weibo attracts users through all kinds of breaking news and spotlight topics, such as the "Japan Earthquake", "Oscar Ceremony", "Boston Marathon Terrorist" and so on. All users can see messages, called Weibos in Chinese, published by concerned users. Given a specific topic of interest, an individual can join the corresponding online social network simply by retweeting (forwarding) or tweeting (posting) the interesting Weibo [15]. To be concrete, we take the empirical data set of the Weibo topic on "Japan Earthquake" and focus on the spatiotemporal dynamics of the user-user retweeting network in terms of characterizing quantities such as the network size, the in-degree and out-degree distributions which correspond to the frequencies of retweeting other or being retweeted by others, and the in- or out-degree correlations. Our main findings are the following: (1) initially the network size increases algebraically with time but it begins to plateau at a critical time when another significant topic of interest emerges; (2) both the in- and out-degrees of the dynamic online-social network follow fat-tailed, approximately algebraic distributions, and (3) the average out-degree is approximately independent of the average in-degree from degree correlation analysis. Based on these results and the rules of online social-network systems, we articulate a theoretic model for the dynamical evolution of these networks. Simulation results of the model agree well with those from the empirical data. Our analysis also suggests a controlled approach to significantly enhancing information spreading on online-social networks.

## Results

The 2011 Japan earthquake is a 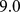 magnitude undersea mega-thrust earthquake occurred on March 11 in the north-western Pacific Ocean near Tohoku, Japan. It was the most powerful earthquake ever hit Japan, which triggered powerful tsunami waves and caused nuclear accidents in the Fukushima Daiichi Nuclear Power Plant complex [16], leading to tremendous loss of human lives and large-scale infrastructure damages. This catastrophe aroused wide concerns and discussions all over the world, especially in China. Since Weibo is the most accessible online social medium in China, a large number of Chinese users joined Weibo to discuss the earthquake and related issues, forming an extremely dynamic user-user retweeting network. We analyze more than 500 thousands Weibo items concerning "Japan Earthquake", starting from the 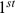 day of earthquake until the 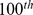 day (defined in Methods). A simple way for a user to join the Weibo social network is to retweet other users' Weibos. The user-user retweeting network can be generated from the data by identifying the retweeting actions among the users. In particular, when a Weibo published by user 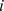 is retweeted by user 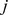, we draw a *directed* link from 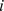 to 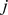. If 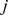 retweets the Weibo published by 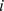 again, another link from 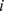 to 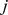 is added, and so on. There can then be duplicate links between any two users in the retweeting network. For the case that a Weibo published by user 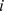 is retweeted by user 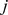, and then retweeted again from 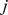 (instead of 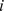) by user 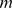, we draw two directed links both from 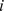 to 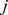 and 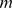. No link from 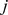 to 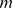 is established since 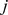 just plays as a intermediary in the associated information spreading process. In the network, a relatively large value of the out-degree indicates that the corresponding user may act as a main source of information, while a large value of the in-degree suggests a high level of retweeting activities of the corresponding user.

### Evolution of the user-user retweeting network

**Figure 1:**
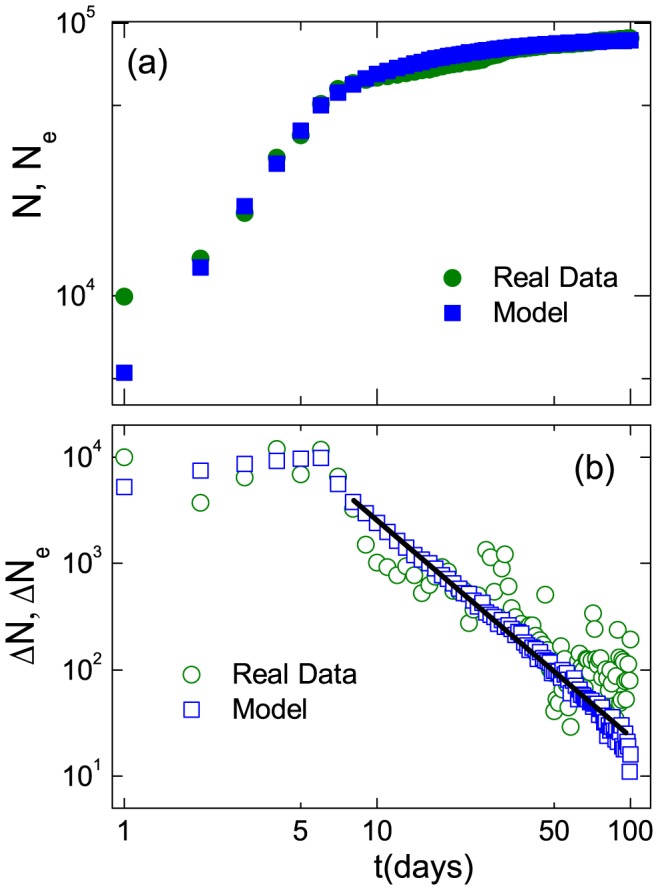
Evolution of user-user retweeting network triggered by the 2011 Japan earthquake. (a) evolution of the cumulative number 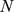 of users with time (green circles), where 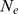 is from our model (blue squares). (b) The corresponding "instantaneous" growth rates 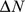 and 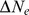.

Figure 1(a) shows the evolution of the number 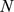 of users involved by retweeting links in days (green circles). We observe that for the initial period of about 7 days, the size of the network increases approximately algebraically with the scaling exponent of about 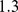. At the critical time 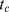, where 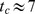, a crossover behaviors occurs, after which the number of nodes increases slowly or plateaus. While in general, an algebraic scaling relation does not permit the definition of some global growth rate, we can still define an "instantaneous" growth rate, the increment 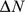 per day. As shown in Fig.1(b), the "instantaneous" growth rate is approximately constant for 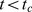, but for 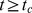, the rate decreases approximately algebraically from about 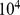 per day to about 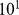 per day at the end of the data duration.

The remarkable change in the temporal behavior of the system on the 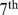 day demands a sensible explanation. By looking into the data further and searching for other medium information about "Japan Earthquake", we find that, at the critical time 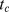, many users switched to discuss the issue of "Salt Rush", which is closely related to "Japan Earthquake." In fact, on the 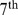 day after the earthquake, a rumor began to spread in Weibo that salt may offer protection against radiation, but the radiation leak from the Fukushima nuclear plant explosion would contaminate sea-salt products [17]. This new topic switched many users' attention from the primary "Japan Earthquake" topic to the "Salt Rush" topic, and for 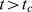 many users stopped discussing the "Japan Earthquake" topic. As a consequence, the instantaneous growth rate for the original topic began to decrease.

### Fat-tailed distribution of in- and out-degrees

**Figure 2:**
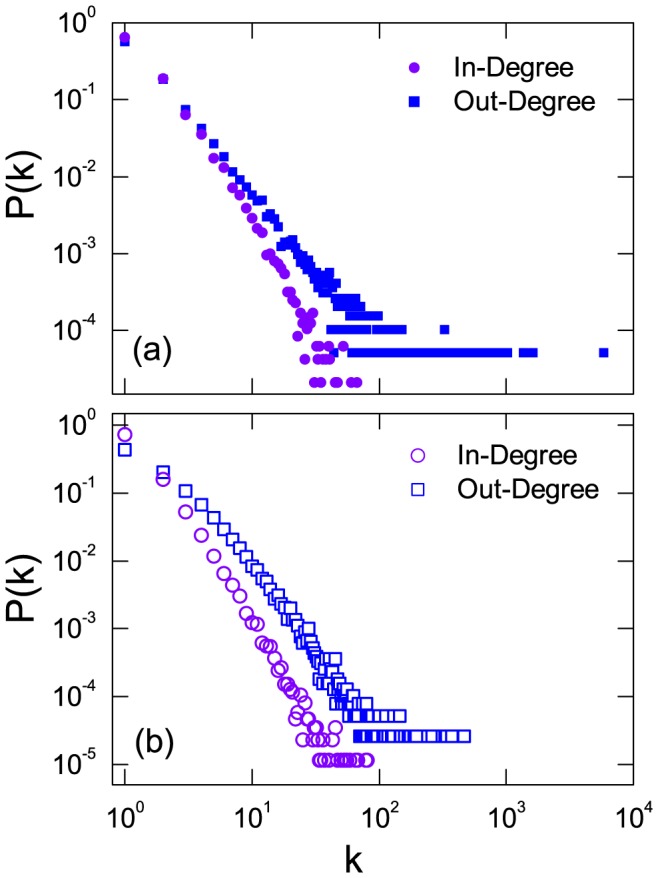
Degree distribution of user-user retweeting network. In- and out-degree distributions of user-user retweeting network generated from real Japan earthquake data (a) and from model (b). The four distribution can be fitted as 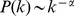 with algebraic scaling exponents 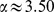, 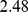, 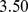, and 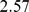 for real in- or out-degree and model in- or out-degree distributions respectively. The distributions were recorded at t = 100 days, and the value of 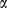 are estimated using the maximum-likelihood estimator [18].

Figure 2(a) displays the distributions of the in- and out-degrees on a logarithmic scale, where we observe approximately algebraic scaling behaviors. Here, the out-degree of user 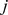, denoted by 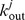, is the total times of 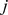
's Weibo(s) being retweeted by other users in the network, and the in-degree of 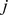, denoted by 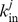, is the retweeting times 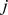 has performed. We note that the algebraic scaling exponents are are 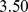 for in-degree and 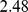 for out-degree distributions. Moreover, the maximum value of in-degree is 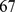 while the out-degree has a much larger maximum value (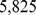). This means that, while the capacity of any individual user to retweet others is limited, users' collective retweeting behavior may congregate, generating superhubs with very large out-degrees. This can be considered as an evidence for the *preferential selection* in the retweeting process introduced by the scheme that Weibo system updates and recommends information.

### Model of user-user behavior network

In the Weibo system, up-to-date topics emerge all the time and are recommended to users through the list of retweeted actions of their *friends* in the order of time. As soon as a new item is added to the recommendation list, one of the early items is removed from the list. This rule stipulates that, when some extreme event occurs, the related topics may rapidly cover the entire recommendation list to attract more users who might not have paid any attention initially. This process could also attract users who are less likely to be interested in the topic. Thus, the number of *potential* users who may join the retweeting network and then become the *enabled* users will increase. This mechanism in fact generates a self-reinforcing (positive feedback) process that makes the messages spread extremely fast initially in the Weibo system. Conversely, this kind of recommendation mechanism may also reduce the number of nodes in the network dramatically when alternative topics emerge. As can be seen from Fig. 1, the event of "Salt Rush" occurring at the 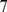
th day after the Japan earthquake is a typical distractive topic with respect to the original earthquake topic. After the distractive topic emerged, the retweeting dynamics associated with the original topic enters into a phase with distinct scaling behaviors.

**Figure 3:**
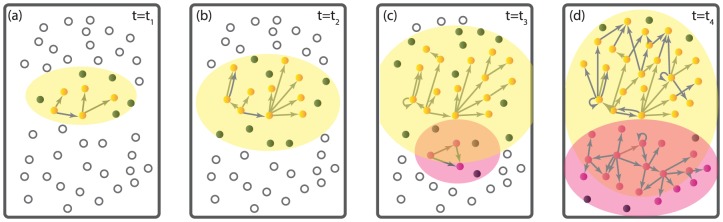
Schematic illustration of the theoretical model generation. (a)-(d) illustrate four instants of the system at 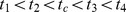, respectively. There exists one topic 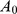 initially, while another topic 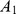 emerges at 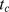. The solid circles (gray, yellow or pink) covered in the yellow (or pink) background areas are the potential users who become interested in the topic 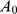 (or 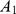), with those colored (yellow or pink) individuals "enabled" by retweeting others. The yellow or pink solid circles respectively are the enabled users of topic 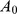 or 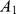.



The sketch map in Fig. 3 briefly illustrates the generation scheme of retweeting network in our model with the aforesaid empirical rules and observations taking into consideration. The dynamical process of retweeting is usually initiated by some primary users' reporting of some specific events. The basic element in the process is the spontaneous retweeting action of some users, *i.e.*, one potential user voluntarily built up a directed link pointed from another user towards him-herself. The final in-degree of each user characterizes its inherent property, *i.e.*, the level of activity in the related topic. The algebraic in-degree distribution signifies the heterogeneity and diversity in the user activities. We are thus led to define the activity level of individual 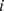 as

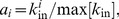
(1)where 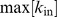 is the maximum in-degree of all users in the system. A potential user 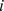 will retweet a related message from others, *i.e.*, to add one in-link, with probability 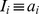 at each time step. As soon as the first in-link is established, the user is *enabled* to behave as a new source of the topic and can be retweeted by others. The *enabled* users are thus those connected to the user-user retweeting network, which can be identified from real data. The probability 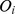 for an enabled user 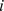 to be retweeted by another potential user, *i.e.*, to add an out-link, is
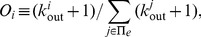
(2)where 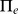 denotes the set of enabled users and the proportional relation is for the reason that, if a user is retweeted by others more frequently, its actions will appear in the recommendation list more times and thus are more likely to be further retweeted.

The temporal evolution of the number of enabled users 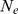 can be obtained analytically. The recommendation mechanism requires that the number of potential users (denoted by 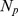) increases with time rapidly in the initial phase of the retweeting process. To gain insights, we first consider the simple case where 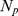 is assumed to be constant. The probability for a potential user 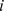 to retweet the topic (*i.e.*, to become enabled) at each time step is 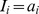 (each user's own level of activity). The probability for user 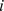 to be enabled before time 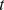 is then 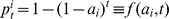. For the case where the users have identical activity level 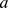, the expectation number of the enabled users at time 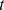 is 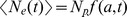, where 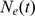 is distributed binomially: 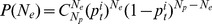. Assuming that the user activity obeys a given probability distribution 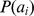, the expectation number of enabled users is
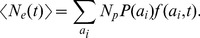
(3)


As can be seen from real data in Fig. 2(a), user activities 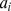 are typically heterogeneous, where the number of retweeted actions performed (the in-degrees) by users ranges from 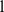 to 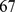 and approximately follows an algebraic distribution 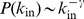, with 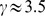.

From the expressions of 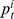 and 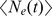, we see that the growth rate of 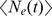 is a monotonic decreasing function of time. However, from Fig. 1, the rate 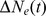 from the real data increases in the initial phase after the network emerges. This discrepancy originates from the simple case assuming constant 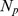 in our probabilistic model, whereas in the real system, 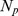 increases rapidly initially as a result of the recommendation mechanism. It is thus necessary to take into account the fact that, at time step 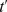, 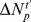 new potential users become aware of the topic from their respective recommendation list in the Weibo page and then retweet with the probability 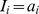. Here, 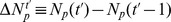 and 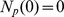. We assume that the time step 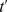 for user 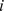 to become aware of the topic is independent of its activity level 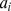. Equivalently, the activity distribution of new potential users at each time obeys the same distribution 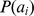. Taking the increment of 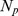 into account, we obtain the expected number of enabled users as
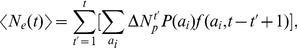
(4)where 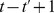 is the duration of the potential users since their awareness of the topic at 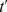. The exact form of the function 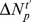 cannot be obtained explicitly, as we can observe from data only increment in the number 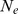 of enabled users. However, we note that the analog of 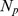 is the *coverage* of a spreading process of the topic associated with the recommendation mechanism, which takes place on the underlying *friendship network* of the Weibo system. We thus have [18], approximately, 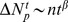, where the parameters 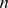 and 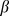 can be obtained by fitting to the real data.

As can be seen from Fig. 1, there is a crossover behavior in the time evolution of 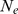 due to the emergence of some alternative topic. For convenience, we name the original topic as 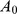 that takes place at 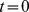 and the new topic as 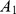 that emerges at 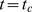. For 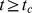, as is illustrated in Figs. 3(c)(d), 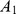 competes for potential users against 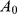. We assume that the basic dynamical process underlying 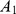 is identical to that of 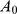. The number of potential users left in 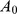 for 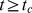 is thus given by 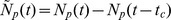, giving rise to a decreasing behavior in the instantaneous growth rate in the number of enabled users.

Our model can be simulated to yield behaviors that reproduce those from the real data. In particular, in the simulation, each user's activity level 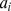 is proportional to its in-degree, whose distribution can be obtained from data. The increment of potential users obtained from data fitting is 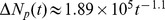. The topic 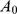 is initially notified by 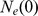 enabled users to trigger the retweeting process [*e.g.*, 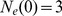]. Results of 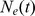 from our model agree well with those from the data, as shown in Fig. 1. The reproduced in- and out-degree distributions are shown in Fig. 2, which again agree with the distributions from the real data.

**Figure 4:**
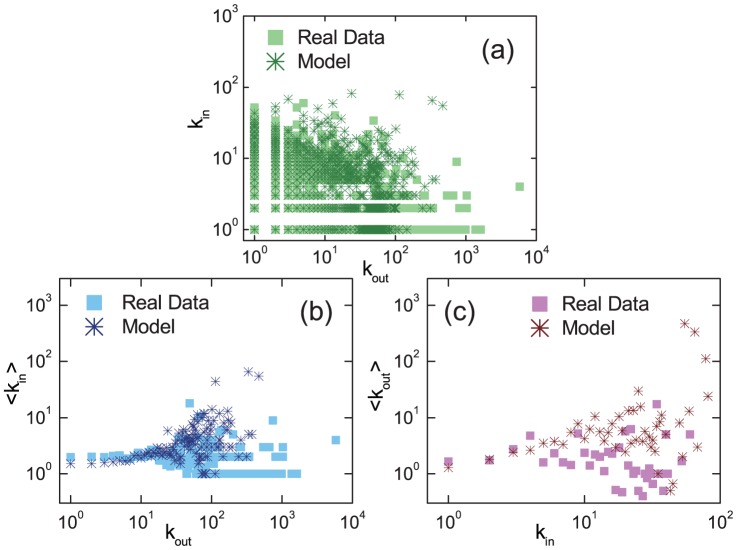
Degree correlations in the user-user retweeting network generated from real data and model. (a) in-degree (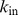) and out-degree (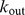) of each user, (b) average in-degree 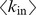 over the users of the same out-degree 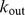, and (c) average out-degree 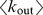 over the users of the same in-degree 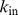.

To further validate our model, we calculate and compare the degree-degree correlation behaviors from the real data and our model. Figure 4(a) plots the out-degree versus the in-degree for all users in the network at time 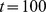. Figures 4(b) and 4(c) show, respectively, the average in-degrees for users having the same out-degrees and the average out-degrees for users with the same in-degrees. The two types of average values are approximately constant but with significant spreads, and the results from our model are qualitatively consistent with those from the real data. The spread can be attributed to the fluctuation due to small amount of large in- or out-degree nodes. Furthermore, we have also calculated the Pearson correlation of the directed networks [19] of the user-user retweeting relation, and the network generated from our model. The four directed assortativity measures from Pearson correlation, i.e., the (in, in), (in, out), (out, in), and (out, out) degree correlations averaged over pairs of neighbor nodes are all found to be around zero.

**Figure 5:**
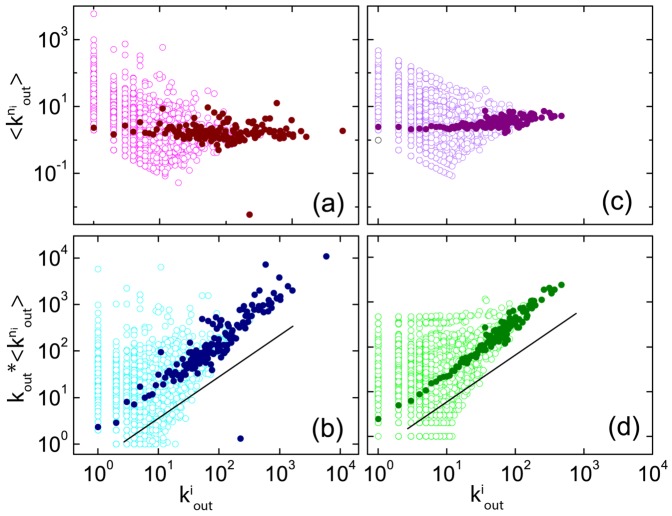
Neighboring out-degrees in user-user retweeting network generated from real data and model. (a) average neighboring out-degree (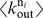) and out-degree (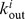) of each user from real data, (b) product between the out-degree and the average neighboring out-degree (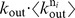) of each user from real data, and (c,d) respective results from model.

What would be an effective way to spread information? In a twitter-like virtual social network, the performance of individual users in the spreading process is determined by their out-degrees 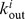 [20], [21]. To select users with larger out-degrees as the sources of spreading would then result in higher coverage in the subsequent time steps. To better understand the spreading process, we plot in Figs. 5(a) and 5(c) the average out-degrees of each user's neighbors, denoted by 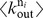, versus the user's own out-degree 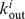, obtained from both real data and from model, respectively, where the solid circles denote the average values of 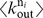 over the users with the same value of 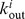. We see that for those users with one given out-degree 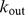, the value of 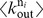 is distributed in a wide interval of about 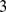 orders of magnitude. However, the average of 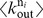 over each 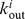 (the solid circle) is approximately constant. Figures 5(b) and 5(d) plot the product of the out-degree and the average neighbor out-degree 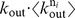, which measures the new information coverage one step after spreading from that particular user. The correlation of 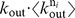 and 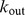 on a logarithmic scale is approximately linear with unit slope both for real data and model. Moreover, the users with larger sum of neighboring out-degrees are those who perform well in the spreading process if they are selected to be the source. The upper-left regions in Figs. 5(b) and 5(d) thus locate the users who are not so popular (small out-degrees) but can spread news efficiently because they have relatively large sums of neighboring out-degrees. These users are the optimal candidates to be controlled for spreading information if a rapid growth of the underlying network is desired.

## Discussion

Online social network systems are becoming increasingly ubiquitous in a modern society. At the present few research are considering their dynamical behavior. Using the approach of empirical-data analysis, we have developed a probabilistic model for the growth dynamics of an important class of such systems: user-user retweeting networks. Our model is capable of reproducing the dynamical and statistical behaviors of the key characterizing quantities such as the growth of the network size, in- and out-degree distributions, and the degree-degree correlations. The development of our model also leads to insights into controlling the information-spreading dynamics on these extremely dynamic networks. Our work represents an initial step in understanding, modeling, and controlling online social network systems, with potential applications not only in social sciences (*e.g.*, for controlling opinion spreading) and commerce (*e.g.*, for developing efficient recommendation algorithms), but also in other disciplines where rapidly time-varying, dynamic networks arise.

## Materials and Methods

### Data collection

We obtain the data from Weibo Open Platform provided by Sina, where we can access the data through accessing Sina API interface freely. The dataset contains 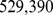 Weibo items with the key word "Japan Earthquake", starting from the day of earthquake (March 11) and ending on June 19, so the duration of the data set is 100 days. The retweeting or retweeted actions of 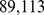 users' were recorded in the dataset. The collected Weibos have the following features: unique message ID of each published Weibo (Mid), unique user ID of each Weibo user (Uid), the publishing time of each Weibo (CreatedAt), the source Weibo's Mid if it is retweeted (rtMid, empty if the Weibo is not retweeted).
